# Supplementary material for: A Novel Fully-Human Potency-Matched Dual Cytokine-Antibody Fusion Protein Targets Carbonic Anhydrase IX in Renal Cell Carcinomas
Source: Front Oncol. 2019 Nov 13;9:1228. doi: 10.3389/fonc.2019.01228 (PMC6863974; doi:10.3389/fonc.2019.01228)
Supplement: Supplementary file 1 [file Data_Sheet_1.PDF]

Supplementary Material

IL2 – 12aa linker – V_H – 14aa linker – V_L – 15aa linker – TNF^{mut}

APTSSSTKKTQLQLEHLLLDLQMILNGINNYKNPKLTRMLTFKFYMPKKATELKHLQCLEEE
LKPLEEVLNLAQSKNFHLRPRDLISNINVIVLELKGSETTFMCEYADETATIVEFLNRWITF
CQSIISTLTGDGSSGGSGGASEVQLLESGGGLVQPGGSLRLSCAASGFTFSSYAMSWVRQAP
GKGLEWVSAIDGSGGSTYYADSVKGRFTISRDNSKNTLYLQMNSLRAEDTAVYYCVKGPPVF
DYWGQGTLVTVSSGGGGSGGGGSGGGGSSELTQDPAVSVALGQTVRITCQGDSLRSYYASWY
QQKPGQAPVLVIYGKNNRPSGIPDRFSGSSSGNTASLTITGAQAEDEADYYCQSSKWSWDPV
VFGGGTKLTVLGSSSSGSSSSGSSSSGVRSSSRTPSDKPVAHVVANPQAEGQLQWLNRAANA
LLANGVELRDNQLVVPSEGLYLIYSQVLFKGQGCPSTHVLLTHTISRIAVSYQTKVNLLSAI
KSPCQRETPEGAEAKPWYEPIYLGGVFQLEKGDRLSAEINRPDYLDFAESGQVYFGIIAL

Supplementary Figure 1: Amino acid sequence of IL2-XE114-TNF^{mut}. Starting from the N-terminus: human IL2, the XE114 antibody in scFv format and human TNF bearing the R431A mutation, corresponding to the position 32 in the soluble form of TNF.

IL2 – 12aa linker – V_H – 14aa linker – V_L – 15aa linker – TNF^{mut}

APTSSSTSSSTAEAQQQQQQQQQQQQHLEQLLMDLQELLSRMENYRNLKLPRMLTFKFYLPK
QATELKDLQCLEDELGPLRHVLDLTQSKSFQLEDAENFISNIRVTVVKLKGSDNTFECQFDD
ESATVVDFLRRWIAFCQSIISTSPQGDGSSGGSGGASEVQLLESGGGLVQPGGSLRLSCAAS
GFTFSLFTMSWVRQAPGKGLEWVSAISGSGGSTYYADSVKGRFTISRDNSKNTLYLQMNSLR
AEDTAVYYCAKSTHLYLFDYWGQGTLVTVSSGGGGSGGGGSGGGGEIVLTQSPGTLSLSPGE
RATLSCRASQSVSMPFLAWYQQKPGQAPRLLIYGASSRATGIPDRFSGSGSGTDFTLTISRL
EPEDFAVYYCQQMRGRPPTFGQGTKVEIKSSSSGSSSSGSSSSGLRSSSQNSSDKPVAHVVA
NHQVEEQLEWLSQWANALLANGMDLKDNQLVVPADGLYLVYSQVLFKGQGCPDYVLLTHTVS
RFAISYQEKVNLLSAVKSPCPKDTPEGAELKPWYEPIYLGGVFQLEKGDQLSAEVNLPKYLD
FAESGQVYFGVIAL

Supplementary Figure 2: Amino acid sequence of mIL2-F8-$mTNF^{mut}$. Starting from the N-terminus: murine IL2, the F8 antibody in scFv format and murine TNF bearing the R448W mutation, corresponding to the position 32 in the soluble form of TNF.

IL2 – <u>12aa linker</u> – V_H – <u>14aa linker</u> – V_L – <u>15aa linker</u> – TNF^{mut}

APTSSSTKKTQLQLEHLLLDLQMILNGINNYKNPKLTRMLTFKFYMPKKATELKHLQCLEEE
LKPLEEVLNLAQSKNFHLRPRDLISNINVIVLELKGSETTFMCEYADETATIVEFLNRWITF
CQSIISTLT<u>GDGSSGGSGGAS</u>EVQLLESGGGLVQPGGSLRLSCAASGFTFSSYAMSWVRQAP
GKGLEWVSAISGSGGSTYYADSVKGRFTISRDNSKNTLYLQMNSLRAEDTAVYYCAKSPKVS
LFDYWGQGTLVTVSS<u>GGGGSGGGGSGGGG</u>SSELTQDPAVSVALGQTVRITCQGDSLRSYYAS
WYQQKPGQAPVLVIYGKNNRPSGIPDRFSGSSSGNTASLTITGAQAEDEADYYCNSSPLNRL
AVVFGGGTKLTVLG<u>SSSSGSSSSGSSSSG</u>VRSSSRTPSDKPVAHVVANPQAEGQLQWLNRAA
NALLANGVELRDNQLVVPSEGLYLIYSQVLFKGQGCPSTHVLLTHTISRIAVSYQTKVNLLS
AIKSPCQRETPEGAEAKPWYEPIYLGGVFQLEKGDRLSAEINRPDYLDFAESGQVYFGIIAL

Supplementary Figure 3: Amino acid sequence of IL2-KSF-TNF^{mut}. Starting from the N-terminus: human IL2, the KSF antibody in scFv format and human TNF bearing the R433A mutation, corresponding to the position 32 in the soluble form of TNF.

IL2 – 12aa linker – V_H – 14aa linker – V_L – 15aa linker – TNF^{mut}

APTSSSTKKTQLQLEHLLLDLQMILNGINNYKNPKLTRMLTFKFYMPKKATELKHLQCLEEE
LKPLEEVLNLAQSKNFHLRPRDLISNINVIVLELKGSETTFMCEYADETATIVEFLNRWITF
CQSIISTLTGDGSSGGSGGASEVQLLESGGGLVQPGGSLRLSCAASGFTFSLFTMSWVRQAP
GKGLEWVSAISGSGGSTYYADSVKGRFTISRDNSKNTLYLQMNSLRAEDTAVYYCAKSTHLY
LFDYWGQGTLVTVSSGGGGSGGGGSGGGGEIVLTQSPGTLSLSPGERATLSCRASQSVSMPF
LAWYQQKPGQAPRLLIYGASSRATGIPDRFSGSGSGTDFTLTISRLEPEDFAVYYCQQMRGR
PPTFGQGTKVEIKSSSSGSSSSGSSSSGVRSSSRTPSDKPVAHVVANPQAEGQLQWLNRAAN
ALLANGVELRDNQLVVPSEGLYLIYSQVLFKGQGCPSTHVLLTHTISRIAVSYQTKVNLLSA
IKSPCQRETPEGAEAKPWYEPIYLGGVFQLEKGDRLSAEINRPDYLDFAESGQVYFGIIAL

Supplementary Figure 4: Amino acid sequence of IL2-F8-TNF^{mut}. Starting from the N-terminus: human IL2, the F8 antibody in scFv format and human TNF bearing the R432A mutation, corresponding to the position 32 in the soluble form of TNF.

IL2 – 12aa linker – V_H – 14aa linker – V_L – 15aa linker – TNF^{mut}

APTSSSTSSSTAEAQQQQQQQQQQQQHLEQLLMDLQELLSRMENYRNLKLPRMLTFKFYLPK
QATELKDLQCLEDELGPLRHVLDLTQSKSFQLEDAENFISNIRVTVVKLKGSDNTFECQFDD
ESATVVDFLRRWIAFCQSIISTSPQGDGSSGGSGGASEVQLLESGGGLVQPGGSLRLSCAAS
GFTFSSYAMSWVRQAPGKGLEWVSAIDGSGGSTYYADSVKGRFTISRDNSKNTLYLQMNSLR
AEDTAVYYCVKGPPVFDYWGQGTLVTVSSGGGGSGGGGSGGGGSSELTQDPAVSVALGQTVR
ITCQGDSLRSYYASWYQQKPGQAPVLVIYGKNNRPSGIPDRFSGSSSGNTASLTITGAQAED
EADYYCQSSKWSWDPVVFGGGTKLTVLGSSSSGSSSSGSSSSGLRSSSQNSSDKPVAHVVAN
HQVEEQLEWLSQWANALLANGMDLKDNQLVVPADGLYLVYSQVLFKGQGCPDYVLLTHTVSR
FAISYQEKVNLLSAVKSPCPKDTPEGAELKPWYEPIYLGGVFQLEKGDQLSAEVNLPKYLDF
AESGQVYFGVIAL

Supplementary Figure 5: Amino acid sequence of mIL2-XE114-$mTNF^{mut}$. Starting from the N- terminus: murine IL2, the XE114 antibody in scFv format and murine TNF bearing the R447W mutation, corresponding to the position 32 in the soluble form of TNF.
